# Effectiveness of techniques for insertion of nasoenteral probe in critical patients: Randomized clinical trial

**DOI:** 10.1097/MD.0000000000033795

**Published:** 2023-05-17

**Authors:** Cintia Galvão Queiroz, Joyce Karolayne dos Santos Dantas, Sara Cristina Matias de Araújo, Mayara Araújo Rocha, Francisco De Cassio de Olivira Mendes, Kleyton Santos Medeiros, Daniele Vieira Dantas, Rodrigo Assis Neves Dantas

**Affiliations:** a Nursing Graduate Program, Federal University of Rio Grande do Norte, Natal, Brazil; b Department of Nursing, Federal University of Rio Grande do Norte, Natal, Brazil; c Institute of Teaching, Research and Innovation, Liga Contra o Câncer, Natal, Brazil; d Department of Nursing, Federal University of Rio Grande do Norte (UFRN), Natal, RN, Brazil.

**Keywords:** bedside placement, coma, critically ill patients, enteral nutrition, gastrointestinal intubation, intensive care, nasally placed feeding tube, nursing, randomized clinical trial, small bowel feeding tube

## Abstract

despite being a common procedure, nasally placed small-bowel feeding tube insertion is not risk-free and can compromise patient safety. Due to the fact that nasally placed small-bowel feeding tube is commonly inserted ‘“blindly,” with the patient head in the neutral position, sometimes the process becomes difficult and traumatic, and may present higher level of complexity in physiological or induced coma and intubated patients. Therefore, adverse events (AEs) route errors can occur during this procedure. This study aimed to determine the effectiveness of different nasally placed small-bowel feeding tube insertion techniques in coma and intubated patients, in comparison with conventional method.

**Methods:**

A prospective, randomized and controlled clinical trial will be carried out with coma and intubated patients admitted to the Intensive Care Unit (ICU). Thirty-nine patients will be randomly divided into 3 groups: group who will have the tube inserted in a conventional manner with the head in the neutral position, group with the head positioned laterally to the right, and, finally, with the head in the neutral position, with assistance of a laryngoscope. The primary endpoint will be: first, second and total attempt success rate; and time required for the first successful attempt and the sum of all attempts. Complications during insertion included tube bending, twisting, knotting, mucosal bleeding, and insertion into the trachea. Patient vital signs will be measured.

**Strengths and Limitations of this Study**

• This is a single-center study, and participants will be recruited from a single hospital, which compromises the generalisability of the findings;
• Blinding of nurses will not be possible;
• The instrument used did not go through the face and content validation process.

## 1. Introduction

According to the World Health Organization, 1 in 10 patients experience unnecessary harm as a consequence of unsafe health care assistance, which in more extreme situations, can cause the patient death.^[1]^ In this context, patient safety is related to a set of activities that aim to mitigate risks to an acceptable minimum, make the error less likely and, when it occurs, reduce the impact^[2]^ prevents the occurrence of adverse events (AEs) or injuries resulting from health procedures.^[3]^

In this context, despite being a common procedure, nasally placed small-bowel feeding tube insertion is not risk-free and can compromise patient safety. Due to the fact that nasally placed small-bowel feeding tube is commonly inserted ‘“blindly,” with the patient head in the neutral position, sometimes the process becomes difficult and traumatic, and may present higher level of complexity in physiological or induced coma and intubated patients, as the tongue displaced backwards blocks the passage through the pharynx. In addition, the piriform sinus and the arytenoid cartilage are considered anatomical obstacles for the nasally placed small-bowel feeding tube passage, which is sometimes bended in the oral cavity due to the inability to swallow and the presence of the endotracheal cannula cuff.^[4]^

Therefore, AEs such as accidental tube displacement, wrong positioning in the respiratory tract, tube obstruction, inadvertent exit, bronchoaspiration and connection route errors can occur during this procedure.^[5,6]^ Among serious and fatal AEs, pneumothorax, aspiration pneumonia and pulmonary hemorrhage stand out.^[7,8]^

There are different nasally placed small-bowel feeding tube insertion methods in critically ill and/or coma patients, which, although less common, have high procedural success rates, but none of them is perfect or universally accepted.^[9,10]^ It is noteworthy that the choice of techniques for this study was based on high success rate, low cost, availability and the lack of need to remove the patient from the Intensive Care Unit (ICU).^[11–14]^

Results favorable to the use of different nasally placed small-bowel feeding tube insertion methods have been described by anesthesiologists.^[11–13]^ Among these, the lateralized head^[5,12]^ and the use of the laryngoscope^[11]^ stand out. Such studies have demonstrated nasally placed small-bowel feeding tube insertion methods that are faster, more effective and that cause less complications to patients. However, it is clear that such techniques are little used by nurses in clinical practice. It is important and necessary for nurses to be able to offer safer care with less risk of complications, based on the best available evidence, through the possibility of revealing the most adequate, effective, fast and safe technique to improve the execution of this procedure.

In a study conducted in Taiwan, 130 insertion attempts were performed. The success rate was 82.7% (86 of 104 patients) on the first attempt and 100% in 5 attempts. In 23 patients the tube deviated to the oral cavity and 3 to the trachea.^[15]^ Verification of vital signs are important to detect clinical deterioration of the patient.^[16]^

We seek to test the following hypothesis: null hypothesis: There are no significant differences between the methods of insertion of the nasally placed small-bowel feeding tube in patients in coma (physiological or induced) and intubated in relation to the rate of success in the first attempt, insertion time and the presence of complications; alternative hypothesis: There are significant differences between the methods of nasally placed small-bowel feeding tube insertion in comatose patients (physiological or induced) and intubated in relation to the rate of success in the first attempt, insertion time and the presence of complications.

Therefore, the study aims to determine the effectiveness of different nasally placed small-bowel feeding tube insertion techniques in coma and intubated patients, in comparison with conventional method.

## 2. Methods

### 2.1. Study design

This is a single center study protocol for a randomized clinical trial to evaluate the effectiveness of different nasally placed small-bowel feeding tube insertion techniques in coma and intubated patients, in comparison with conventional method. This study will adhere to the Standard Protocol Items for Randomized Trials^[17]^ and the Consolidated Standards of Reporting Trials.^[18]^ The data collection stage is ongoing.

### 2.2. Study setting

Recruitment will be from patients admitted in an ICU of a tertiary hospital from the state of Rio Grande do Norte, Brazil.

### 2.3. Population

Physiological or induced coma and intubated patients admitted to the ICU in will be included.

### 2.4. Eligibility criteria

The study will include patients in physiological coma (score between 3 and 8 on the Glasgow Coma Scale) or induced coma (score between 5 and 6, slow response to stimuli and deep sedation, respectively), on the Ramsay scale, submitted to invasive mechanical ventilation, indication of nasoenteral tube under medical prescription, aged 18 years or older. It is noteworthy that patients who lost the nasoenteral tube due to inadvertent exit or obstruction will also be included.

Exclusion criteria were: patients with coagulopathy (abnormal prothrombin time, partial thromboplastin time and platelet disorders); nasal stenosis; upper respiratory tract anomalies; esophageal disorders (esophageal hiatus hernia, esophageal varices, esophageal or nasopharyngeal carcinoma); skull base fracture; teeth with mobility and history of difficult intubation. In order to verify such information, medical records were consulted before the procedure was performed.

### 2.5. Recruitment

**Figure 1.**
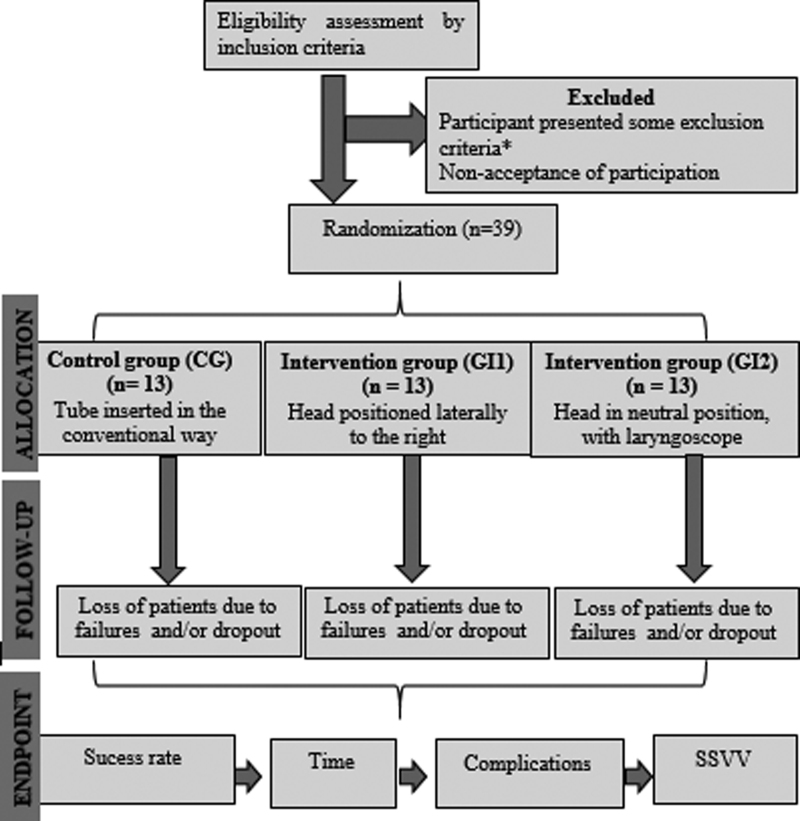
Flowchart of inclusion, allocation, follow-up procedures and study endpoints. Natal/RN, 2021. *Patients with coagulopathy (abnormal prothrombin time, partial thromboplastin time and platelet disorders); nasal stenosis; upper respiratory tract anomalies; esophageal disorders (esophageal hiatus hernia, esophageal varices, esophageal or nasopharyngeal carcinoma); skull base fracture; teeth with mobility and history of difficult intubation.

The study flowchart is described in Figure 1. Patients will be recruited upon the need for nasoenteral tube insertion the ICU. At the opportunity, the eligibility criteria for participation in the research will be applied. Subsequently, as critical patients admitted to the ICU are unable to consent, consent will be obtained from legal guardians prior to the intervention. Each research participant will have to fill out the Free Informed Consent Form, with the reading and signature of the respective family member legally responsible for the patient.

### 2.6. Allocation

In this study, only data analysts will be blinded to the treatment group to which participants were randomized. However, resources will be used to minimize, as much as possible, biases in research results.

For this, there will be a collaborating researcher (P1), who will be responsible for the allocation and randomization of patients prior to data collection using computer-generated random numbers, to be carried out through the “Research Randomizer” website (https://www.randomizer.org), in addition to the application of the collection instrument, verification of the insertion time and annotation of the vital signs and application of the collection instrument, as well as the reading of the insertion X-ray, to be performed immediately after the insertion of the nasally placed small-bowel feeding tube. Three nurses (E1, E2, E3) will be in charge of applying any of the programmed techniques. It is noteworthy that nurses responsible for applying the techniques are from the service where the data collection will be carried out and work on the day and night shifts and will be unaware of topics to be evaluated. Although the nurses are aware of the method that will be used for the insertion of the tube, they will not be aware of the objectives of the study. This way, only the investigation team will know which technique was used in each patient, and patients will not know due to their clinical condition.

To minimize biases, it is noteworthy that, prior to the data collection stage, nurses were trained in relation to the techniques applied to familiarize with the procedure, as this fact can influence the success rate and the time required for insertion. To this end, a previously elaborated script will be strictly followed for each of the techniques, with nurses being trained in theory and laboratory practice. Materials and monitoring equipment used will also be standardized. The PHILIPS CM10 Efficia multiparameter will be used to monitor all patients and the nasally placed small-bowel feeding tube (EMBRAMED).

### 2.7. Intervention

In all patient groups, a 120 cm 12 French gauge (1 French = 0.33 mm) nasally placed small-bowel feeding tube was standardized for use in the institution where the study will be carried out. The length of the tube needed to reach the jejunum will be assessed prior to insertion and measured by placing the nasally placed small-bowel feeding tube from the earlobe to the xiphoid process and then down to the umbilicus (EXU method).^[19]^ Before insertion, 3 milliliters of 2% xylocaine will be applied to the patient nostril, selecting the one with no nasal obstruction or deviated septum. In each group, the nasally placed small-bowel feeding tube will be passed through the patient nostril, posteriorly along the floor of the nose and advanced to reach the measured length.

### 2.8. Endpoint

#### 1.2.8. Primary outcome.

The success rate on the first attempt will be considered as the dependent variable. Insertion success will be defined as the successful passage of the tube on the first attempt and confirmed by radiography of the correct anatomical positioning of the tube distal tip, which will be performed immediately after the procedure. If the nasally placed small-bowel feeding tube is not successfully inserted, it will be considered a failure and recorded as an unsuccessful attempt. Subsequently, new attempts will be performed.

The number of attempts required for successful insertion will be recorded for each patient. It should be noted that if more than 3 attempts are required, the procedure will be declared unsuccessful and data will be used to count failures. In these cases, the researchers may use another method of free choice for nasally placed small-bowel feeding tube insertion.

#### 2.2.8. Secondary outcome.

Secondary endpoints will be time required and associated complications. The time required will be recorded from the nasally placed small-bowel feeding tube insertion from the nostril to the calculated length of the tube with the aid of a stopwatch. The insertion times for each attempt will be counted and, in case of failures, the time required for each attempt will be added together.

In addition, AEs associated with the insertion techniques (bleeding of the visible mucosa, nasal mucosa edema, in addition to tube bending, twisting, knotting and insertion into the trachea visible on X-ray) will be recorded. It is noteworthy that the occurrence of bleeding will be considered as visible bleeding in the tube extension and nasal or oral mucosa. The analysis of such AEs will be performed immediately at the end of the procedure until radiological confirmation of the tube positioning and previous removal of the guide wire. The radiograph will be performed on the patient bed and the patient will be kept in the same position, in the supine position. If the patient shows signs of respiratory distress during insertion, the procedure will be stopped.

### 2.9. Sample size

The sample size calculation for this study was established using the G Power software, version 3.1.9.2 (available at: http://www.gpower.hhu.de/), which considered Cohen effect size of 0.80, power test of 0.80 and significance level of 5% (*P* < .05). Thus, a total sample of 39 patients was obtained, with 13 participants assigned to the Control Group (CG) and 26 to each Intervention Group (GI1 and GI2).

### 2.10. Randomization and masking

Thirty-nine patients will be randomly divided into 3 groups. Patients allocated in the control group (CG) will have the tube inserted in the conventional way, that is, the head is kept in the neutral position, while the tube is gently inserted through one of the nostrils, without any maneuver or instrument.

Patients selected for the first intervention group (GI1) will have their heads turned to the right lateral position. This way, the tube will be inserted through the selected nostril without any neck maneuver or instrument.

For patients selected for the second intervention group (GI2), a conventional adult laryngoscope will be orally inserted. The tracheal tube and tongue will be raised to provide the best view of the larynx area. Thus, after viewing the piriform sinus or the esophagus, the tube will be inserted into the patient nostril and advanced to the esophagus under direct visualization. The patient head will be held in the neutral position and the blade will be gently lifted to avoid excessive neck extension.

### 2.11. Data collection

A data collection printed form containing variables about patients will be used, namely: Identification (bed; name initials; age; sex; date of birth); Clinical data (cause of admission; estimated weight, height and body mass index); Level of consciousness or Ramsay scale data, including type of drug used and sedation duration; Endotracheal tube number and cuff pressure; Data regarding the technique used (technique; number of attempts; required time in seconds; complications, if any); Parameters to be evaluated (heart and respiratory rate, blood pressure and oxygen saturation) before, during and after nasally placed small-bowel feeding tube insertion using each of the techniques; Confirmation of the correct nasally placed small-bowel feeding tube positioning by chest and abdomen radiography in bed.

Table 1 shows the detailed study stages, with the Standard Protocol Items for Randomized Trials schedule of enrollment, interventions, and assessments.

**Table 1:** Description of the study stages: P1: Day 1—request for nasally placed small-bowel feeding tube insertion in the patient and intervention. F1: evaluation after the procedure until X-ray is performed.

Study period					
			Post-allocation: Allocation, interventions, follow-up		
Timepoint	Enrollment	Baseline	Allocation	P1 intervention	Follow-up (F1)
Enrollment:					
Eligibility criteria	X				
Recruitment	X				
Initial assessment	X				
Informed consent	X				
Allocation			X		
Interventions:					
Insertion in a conventional manner (control group)				⟷	X
Insertion with the head turned to the right side (intervention group)				⟷	X
Insertion with laryngoscope (intervention group)				⟷	X
Assessments:					
Identification, clinical data, level of consciousness, SSVV, among others		X			

### 2.12. Data collection and management

The principal researcher will be responsible for recruiting participants and applying the data collection instrument. Three nurses will be responsible for applying the intervention in the control and intervention groups, without knowing what will be evaluated.

The participation of patients will be carried out on a voluntary, nonprofit basis and will occur upon reading and signing the Free and Informed Consent Form by the family member, which demonstrates that the individual accepts to participate in the research, explaining all stages, objectives, risks and benefits of the research. It is possible for participants to dropout at any stage of the research without prejudice to treatment and without suffering penalties or judgments of any kind.

The researchers will ensure that the anonymity of participants is protected and their data will remain confidential so that their identities and any type of identity information is protected. Two copies of the consent form will be provided, both signed by a family member and responsible researchers.

The results of this research will be presented at meetings or publications; however, the identity of participants will not be revealed. Original documents and files will be kept at the test sites for 15 years. The principal investigator is responsible for storing data and files and will have access to the final set of data and dissemination.

### 2.13. Management of losses and dropouts

Losses and dropouts will be carefully recorded, ensuring the reliability of the research. If any participant in the sample withdraws from participating in the study, he/she will be removed from collection and another participant will be included.

### 2.14. Data extraction and statistical analysis

Data will be analyzed using the Statistical Package for Social Science software, version 18.0 (SPSS Version 20.0), where descriptive and inferential analyses will be carried out, and results will be presented through tables, charts and figures. To evaluate variables, the Chi-square test (χ²) or Fisher test or ANOVA will be used. Other statistical tests that may be necessary will also be used. Significance level of 5% (*P* = .05) will be adopted.

### 2.15. Patient and public involvement

All the patient information will be anonymously analyzed and processed. Patients and/or the public were not involved in the design, or conduct, or reporting, or dissemination plans of this research.

## 3. Discussion

The analysis of the effectiveness of different nasoenteral tube insertion techniques in coma (physiological or induced) and intubated patients is initially necessary due to the difficulty of inserting small-bowel feeding tube in a blind manner, with the patient head in the neutral position. The high rate of complications resulting from the small-bowel feeding tube insertion due to the absence of a proven safer method poses a risk to the patient.

A randomized clinical trial emphasizes that the small-bowel feeding tube insertion is often a challenge for ICU professionals. Therefore, the use of instruments that help through visualization was also reported as a facilitator for tube insertion and proper small-bowel feeding tube positioning in critically ill patients, such as laryngoscopes and videolaryngoscopes. Radiological and endoscopic techniques and methods guided by electromagnetism and fluoroscopy will also be used.^[13]^

Therefore, it is important and necessary to offer safer care with less risk of complications based on the best available evidence for the possibility of revealing the most adequate, effective, fast and safe technique to improve the performance of this procedure.

In addition, there is the possibility of characterizing nursing care based on new techniques in order to be used in clinical practice and to expand the knowledge base and skills of nurses in managing the small-bowel feeding tube insertion in critically ill patients, being an accessible and low-cost technology that can be used by nurses, streamlining work processes and contributing to adequate, safe and quality care.

Results favorable to the use of different nasoenteral tube insertion methods have been described by anesthesiologists.^[14–16]^ Among these, the lateralized head^[4,15]^ and the use of the laryngoscope^[4]^ stand out. Such studies have demonstrated small-bowel feeding tube insertion methods that are faster, more effective and that cause less complications to patients.

However, it is clear that such techniques are little used by nurses in clinical practice, in addition to a gap related to the deficiency of experimental studies with high level of evidence in relation to new small-bowel feeding tube insertion techniques to aid decision-making in clinical practice, which justifies the conduction of the present study.

## Author contributions

**Conceptualization:** Cintia Galvão Queiroz, Mayara Araújo Rocha, Kleyton Santos Medeiros, Daniele Vieira Dantas, Rodrigo Assis Neves Dantas.

Formal analysis: Cintia Galvão Queiroz, Francisco De Cassio de Olivira Mendes, Kleyton Santos Medeiros, Daniele Vieira Dantas, Rodrigo Assis Neves Dantas.

Investigation: Cintia Galvão Queiroz, Joyce Karolayne dos Santos Dantas, Sara Cristina Matias de Araújo.

Methodology: Cintia Galvão Queiroz, Mayara Araújo Rocha, Francisco De Cassio de Olivira Mendes, Kleyton Santos Medeiros, Daniele Vieira Dantas, Rodrigo Assis Neves Dantas.

Supervision: Daniele Vieira Dantas, Rodrigo Assis Neves Dantas.

Validation: Mayara Araújo Rocha, Francisco De Cassio de Olivira Mendes, Kleyton Santos Medeiros, Rodrigo Assis Neves Dantas.

Visualization: Mayara Araújo Rocha.

Writing – original draft: Cintia Galvão Queiroz, Joyce Karolayne dos Santos Dantas, Sara Cristina Matias de Araújo, Francisco De Cassio de Olivira Mendes, Rodrigo Assis Neves Dantas.

Writing – review & editing: Cintia Galvão Queiroz, Joyce Karolayne dos Santos Dantas, Sara Cristina Matias de Araújo, Mayara Araújo Rocha, Francisco De Cassio de Olivira Mendes, Kleyton Santos Medeiros, Daniele Vieira Dantas, Rodrigo Assis Neves Dantas.
